# Oncologic long-term outcome of single-incision laparoscopic surgery (SILS) for colorectal cancer

**DOI:** 10.1007/s00384-021-03902-0

**Published:** 2021-03-16

**Authors:** Andreas D. Rink, Vitaly Golubev, Boris Vestweber, Claudia Paul, Hauke Lang, Karl-Heinz Vestweber

**Affiliations:** 1 Department of General, Visceral and Transplant Surgery, University Medicine of Johannes Gutenberg-University, Langenbeckstr 1, 55131 Mainz, Germany; 2 Department of General, Visceral and Thoracic Surgery, Leverkusen General Hospital, Am Gesundheitspark 11, 51375 Leverkusen, Germany; 3 Department of Surgery, King Edwards VII Memorial Hospital, 7 Point Finger Rd,, Paget DV 04 Hamilton, HM DX, Bermuda

**Keywords:** Single access surgery, Minimally invasive surgery, Matched pair, Colon cancer, Rectal cancer

## Abstract

**Purpose:**

Single-incision laparoscopic surgery (SILS) has been introduced as a less invasive alternative to multi-port laparoscopic surgery (MLS). MLS is widely accepted for the treatment of colorectal cancer, but there remains minimal evidence for the use of SILS. Thus, we compared both short- and long-term outcomes of SILS and open surgery (OS) in matched cohorts of colorectal cancer patients.

**Methods:**

Some 910 patients had colorectal resections for cancer between 2006 and 2013, and 134 of them were operated on using SILS. Eighty of these SILS patients were compared to a cohort of patients who had open surgery that were matching in tumour stage and location, type of resection, sex, age and ASA Score. Disease-free survival at 5 years (5y-DFS) was the primary endpoint; morbidity and hospitalization were secondary parameters. The role of surgical training in SILS was also investigated.

**Results:**

Clavien Dindo ≥ IIIb complications occurred in 13.8% in both groups. 5y-DSF were 82% after SILS and 70% after OS (*p* = 0.11). Local recurrence after rectal cancer tended to be lower after SILS (0/43 (SILS) vs. 4/35 (OS), *p* = 0.117). Length of stay was significantly shorter after SILS (10 vs. 14 days, *p* = 0.0004). The rate of operations performed by surgical residents was equivalent in both groups (44/80 (SILS) vs. 46/80 (OS), *p* = 0.75).

**Conclusion:**

The data demonstrates that SILS results in similar long-term oncological outcomes when compared to open surgery as well as morbidity rates. The hospital stay in the SILS group was shorter. SILS can also be incorporated in surgical training programmes.

## Background

Minimally invasive surgery has been proven as an accepted alternative to open surgery for the treatment of colorectal cancer [1]. At least oncological non-inferiority has been demonstrated for both colon and rectal cancer surgery, and for patients operated on laparoscopically, earlier postoperative recovery has been demonstrated repeatedly [2, 3]. Single-incision laparoscopic surgery (SILS) has been introduced as a subtype of minimally invasive surgery that further minimizes the surgical trauma [4–6]. The entire operation is performed through one incision which is also used to harvest the surgical specimen in most cases. Some studies have demonstrated additional benefits of this less invasive approach in comparison to multiport laparoscopic surgery (MLS). The benefits are faster postoperative recovery, shorter length of hospital stay, less pain and better cosmetic outcomes [7]. We have published our initial data with intraoperative and early postoperative outcomes in matched cohorts of patients operated on with SILS versus conventional open surgery. The results showed lower morbidity rates and shortened length of hospital stay with SILS patients while the oncological outcomes were equivalent [8]. It is important to note that the effectiveness of SILS with oncological patients for the treatment of colorectal cancer in comparison to open or laparoscopic surgery has not been studied sufficiently and there is no long-term data available from well-designed comparable trials.

In order to investigate the oncological safety of SILS for the treatment of colorectal cancer, we performed a matched comparison of early postoperative recovery and long-term oncological outcomes of patients treated with SILS or with open surgery.

## Patients and methods

SILS was introduced at Klinikum Leverkusen for the treatment of benign colorectal diseases in July 2009. SILS experienced consultants expanded their knowledge of this new technique on a selection of patients with colorectal malignancy as an alternative to conventional open surgery. Patients with expected T1–3 tumours were deemed eligible for SILS. The criteria for SILS excluded patients with a body mass index of > 45 kg/m^2^, a history of major abdominal surgery with a midline incision and an American Society of Anaesthesiology (ASA) stage > III. All oncological patients operated on up until December 2013 with SILS resection for colorectal adenocarcinoma were included in the analysis and the data was recorded prospectively.

A reliable control group was created out of a cohort of 756 patients who had open surgery for colorectal cancer at Leverkusen General hospital between January 2006 and December 2013. In order to reduce biases, we created a control group matching for the following covariates.

- Tumour localisation

- Right colon
- Transverse colon
- Left colon
- Rectum

- Operative procedure
  - Right hemicolectomy
  - Transverse colectomy
  - Left hemicolectomy or oncologic sigmoid colectomy
  - Anterior rectal resection with partial mesorectal excision (PME)
  - Sphincter-preserving total mesorectal excision (TME)
  - Abdominoperineal excision

- Tumour stage according to the Union internationale contre le cancer (UICC) - classification using the main and subgroups
- Sex
- American Society of Anesthesiologists Score (ASA Score)
- Age category
  - 20–40 years
  - 41–60 years
  - 61–80 years
  - > 80 years

Data from the control patients were collected retrospectively from the clinical information system and the patients’ charts.

The primary end point of the study was disease-free survival at 5 years after surgery (5-yDFS). Secondary end points were:

- Overall survival at 5 years
- Oncologic surrogate parameters (lymph node harvest, r-status, distal resection margins in rectal cancer)
- Morbidity and mortality at 30 days
- Length of hospital stay

### Primary surgeons’ experience

The level of surgeon’ experience was taken into consideration to investigate potential expert bias. This was done by the procedures being categorised into the following three types.

#### Expert level procedures

The primary surgeon was an experienced colorectal surgeon with at least 20 personally performed SILS procedures.

#### Advanced training operation

The primary surgeon was a board-certified general or colorectal consultant surgeon with limited experience in SILS. The operation was done under continuous supervision by an experienced colorectal consultant with SILS experience (SILS group) or not necessarily with SILS experience (control group).

#### Teaching operation

A surgical resident performed the operation under immediate supervision of an experienced colorectal consultant (open surgery) or an experienced colorectal surgeon with experience of at least 20 personally performed SILS procedures (SILS)

### Surgical technique

Standard bowel preparation was performed using 30 g of magnesium sulphate. All procedures were performed under general anaesthesia. A selection of patients received epidural anaesthesia for additional pain control. Patients with left-sided colon or rectal cancer were placed in the lithotomy position and those with cancer at other sites were placed supine. A 3–4-cm umbilical vertical incision was made. In patients likely to require a temporary or permanent stoma, the incision was made at the site of its proposed stoma location. The SILS port was inserted through a wound retractor/protector (Alexis Applied Medical Research Corp., Rancho Santa Margarita, CA) and inserted through the umbilicus. A capnoperitoneum of 14 mmHg was established. Dissection was performed with standard straight laparoscopic instruments (Olympus Key-Med, Southend-on-Sea, Essex, UK) or LigasureTM 5-mm blunt tip sealer (Covidien). A 50-cm-long 30°, 5-mm laparoscope (Karl-Storz GmbH & Co. KG, Tuttlingen, Germany) was used in all cases. From 2009 to 2012, the vessels were dissected with a lateral to medial approach. Starting from 2013, this dissection was preferred to be performed in a medial to lateral approach. The ileocolic and right colic vessels were divided intracorporeally for right colonic resections. In these right colonic resections, the colon was divided in most patients intracorporeally using Endo-GIA (Covidien, Neustadt, Germany) but in all cases the anastomosis was done extracorporeally via stapling or sutured by hand. For left colonic resections, the inferior mesenteric vein was divided at the level of the tail of the pancreas. The inferior mesenteric artery was divided at a point close to the aorta preserving the branches of the superior mesenteric plexus. For left colonic and sphincter-preserving rectal resections, an Endo-GIA was inserted directly through the SILS port without using a trocar and the rectum was divided at the appropriate level. The specimen was extracted via the SILS port incision leaving the wound retractor/protector in place and a double-staple anastomosis was performed. In patients with large tumours or obesity, the incision was expanded to enable a safe harvest of the specimen to avoid tumour contamination. Patients having an abdomino-perineal resection for advanced low rectal cancer had the laparoscopic dissection performed to the mid-rectum so the plane between the rectum and the levator muscle was avoided. The patient was then turned into the prone position and a cylindrical excision was performed as described by Holm et al. [9]. An additional trocar was used if technical difficulties were encountered. If the SILS resection was not possible to perform safely, the procedure was converted to open surgery.

All open operations were done through midline incision.

### Follow-up

Follow-up was performed according to the German guidelines. All patients intended to have SILS procedures had prospectively been collected in a database (IBM SPSS). The follow-up data was also collected prospectively by personally performed, telephone based, or written post-mailed questionnaires at least once a year. The data of the control patients was collected retrospectively. The follow-up examinations (ultrasound, colonoscopy, clinical examination, tumour markers) were partially done by medical practitioners out of the hospital. In these cases, the reports on the findings were requested upon approval of the patients.

### Ethics

Written informed consent concerning the surgical techniques was obtained from all individuals included in this trial. The patients treated with SILS were specifically informed about the new technique and the limited evidence of its use for the treatment of colorectal cancer.

### Statistics

All data were collected and analysed with IBM SPSS Statistics 25. Computer-assisted matching cluster filter applications using Visual Basic 6.0 (Microsoft ADODB Technology) were performed. The data is presented as median and range. The chi-square test was used to compare categorial, and the non-parametric Wilcoxon test for continuous, data, respectively. Survival data were compared with the Kaplan-Meier method and log rank tests were performed for statistical evaluation. A *p* < 0.05 was considered statistically significant. Data analysis was done on an intend-to-treat basis.

## Results

Between January 2006 and December 2013, a total of 910 patients had surgery for colorectal malignancy at Leverkusen General Hospital. The SILS technique was used in 154 cases. In 16 of these cases, SILS was used to perform local excisions or segmented resections for early tumours or for palliative patients. Three of the remaining 138 cases did not have colorectal adenocarcinoma. One patient had surgery for cancer originating from a long standing fistula. Eighty of the remaining 134 patients with oncological SILS resections for colorectal adenocarcinoma perfectly matching control patients regarding the criteria mentioned above (age category, sex, tumour location, tumour stage, surgical procedure, ASA Score) could be identified among the cohort of 756 patients who had open surgery. Forty-six of the patients in each group had rectal cancer and 34 colon cancer. The numbers of patients that failed to follow-up 60 months after surgery were 5/80 (6.2%) in the SILS and 2/80 (2.5%) in the control group. After 36 months, it was one patient in each group (1.2%). Overall, the mean follow-up time was 58.8 months in the SILS and 74.3 months in the control group.

The demographics for both groups are presented in Table 1. The patients’ body mass index was not used for matching and there was no significant difference in BMI (*p* = 0.9) but the proportion of patients with a BMI > 30 kg/m^2^ tended to be higher in the control group (*p* = 0.09). An equivalent number of patients had adjuvant treatment.

**Table 1:** Basic characteristics

		SILS	Control	*p*
Sex (f/m) (*n*)		20/60	20/60	1
Age		67.7 (43–87)	66.1 (35–85)	0.168
BMI (kg/m^2^)		25.4 (19–34)	27.3 (18–43)	0.9
Obesity (BMI ≥ 30 kg/m^2^) (*n* (%))		10/80	23/80	0.09
ASA Score I/II/III/IV/V (*n*)		8/40/29/3/0	8/40/29/3/0	1
Tumour location	Coecum	3	3	1
	Ascending colon	8	8	
	Transverse colon	1	1	
	Descending colon	2	2	
	Sigmoid colon	20	20	
	Upper rectum (12–16 cm)	19	19	
	Mid rectum (> 6–12 cm)	16	16	
	Lower rectum (0–6 cm)	11	11	
UICC stage*	0	4	4	1
	I	20	20	
	IIA	18	18	
	IIb	1	1	
	IIIA	5	5	
	IIIB	24	24	
	IIIC	4	4	
	IVA	4	4	

*Postoperative pathologic staging

Table 2 displays the details of the procedures performed in both groups, the primary surgeon’s experience and the early postoperative outcomes. The length of stay was significantly shorter after SILS when compared to open surgery. Morbidity tended to be higher in the control group. However, the number of Clavien-Dindo grade IIIb or higher complications were identical with 11/80 severe complications (13.8%) in each group. There were significantly more expert level operations in the SILS group. However, a similar proportion of the procedures were teaching operations in both groups.

**Table 2:** Procedures and early postoperative outcome

		SILS (*n* = 80)	Control (*n* = 80)	*p*
Type of surgery				
Right hemicolectomy		11	11	1
Transverse colectomy		1	1	
Left hemicolectomy or sigmoid colectomy		19	19	
Anterior rectal resection (PME)		21	21	
Low anterior resection (TME)		22	22	
Abdomino-perineal resection		7	7	
Other technical aspects				
Duration of surgery (min)		227 (97–500)	192 (59–543)	0.1
Conversion to open surgery		11 (13.8%)	-	-
One additional trocar		5 (6.2%)		-
Surgeons’ experience (expert/advanced training/teaching)		30/6/44	16/18/46	0.002
Morbidity				
Patients with at least one complication		27/80	43/80	0.274
Classification of morbidity according to Clavien–Dindo	I	3	10	
	II	9	19	
	IIIA	4	3	
	IIIB	9	9	
	IV	1	1	
	V	1	1	
Length of stay (days)		10 (4–57)	14 (1–81)	0.0004

**Fig. 1:**
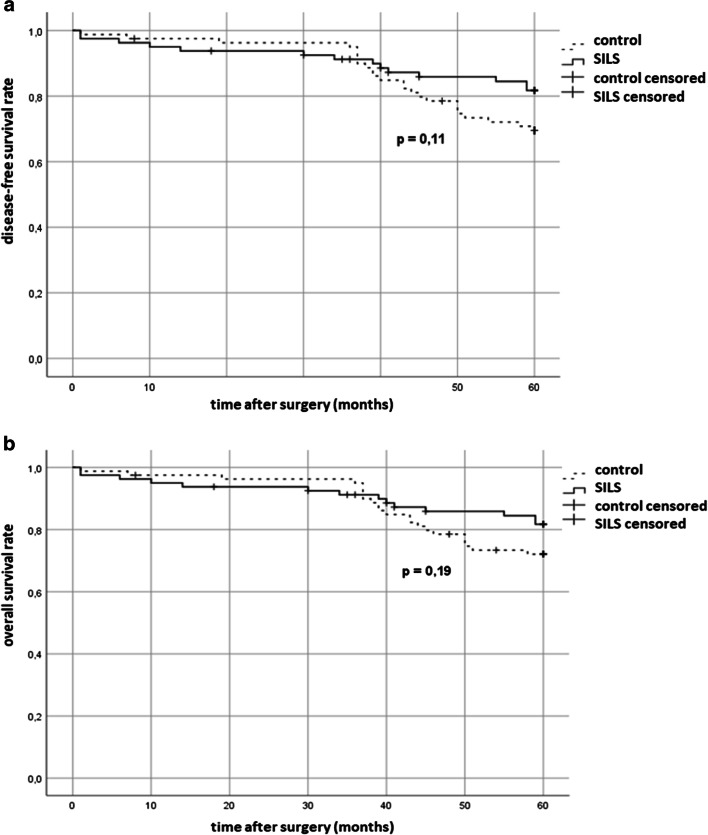
a Disease-free survival after SILS or conventional surgery for colorectal cancer. **b** Overall survival after SILS or conventional surgery for colorectal cancer

Oncological parameters were equivalent in both groups. There was no significant difference in the use of adjuvant therapy (see Table 3). The oncological long-term outcome is illustrated in Fig. 1a and b. The 5-year disease-free survival were 82% in the SILS and 70% in the conventional group (*p* = 0.11). The estimated overall survival at 5 years were 82% after SILS and 72% after conventional surgery (*p* = 0.19). Oncological surrogate parameters were distributed equivalently between the two groups. Local recurrence occurred in 4 out of 45 rectal cancer patients in the control group but none in the 43 patients in the SILS group (*p* = 0.117). There was no patient with documented peritoneal carcinomatosis in either group.

**Table 3:** Oncologic surrogates and adjuvant treatment

Lymph node harvest	20 (7–41)	21 (1–61)	0.731
Distal resection margin [cm] (rectal cancer)	5 (1.3–7)	5 (2–6.5)	1
R0-resection	80/80	80/80	1
Postoperative adjuvant chemotherapy	48/80	45/80	0.633

## Discussion

Minimally invasive surgery is a well-established treatment for colorectal cancer. Most randomized trials comparing MLS and open surgery resulted in similar oncologic results. The MLS postoperative recovery was faster, the complications less frequent and hospitalisation shorter [2, 3]. The COLOR-2-trial has clearly proven the minimally invasive approach for rectal cancer is comparable, while the subgroup of patients with stage III cancers displayed a superior disease-free survival rate [10]. Two studies initially failed to prove non-inferiority as long as oncologic surrogate parameters were investigated [11]. However, the clinical outcome data finally proved to be equivalent [12, 13]. Thus, minimally invasive surgery for the treatment of rectal cancer has clearly been shown to be successful.

The single-incision approach is the least invasive version of laparoscopic surgery. SILS is considered to further reduce postoperative pain and improve recovery when compared to open surgery [7]. However, the technique can be technically challenging. Most surgeons use standard straight laparoscopic instruments. The lack of triangulation and the need for parallel positioning can make adequate dissection challenging. A total of five randomized controlled trials comparing SILS versus MLS for the treatment of colorectal cancer have been published [14–18]. In summary, these trials demonstrate that SILS for colorectal cancer can be used safely but has no clear advantage over MLS.

We directly compared SILS with open surgery and found that SILS was associated with a significant shorter hospital stay; however, the median length of stay was long in both groups. The primary reason for this is the patients’ expectation of hospitalisation in Germany and their health insurances completely cover all expenses of in-hospital stay. Regardless, the less invasive procedure resulted in a significant reduction of hospitalisation. Complications occurred slightly less frequently in the SILS group. This finding is consistent with the result of our earlier published matched pair comparison of patients treated for colorectal cancer by SILS versus open surgery [8]. The subgroup of patients with colon cancer had fewer wound complications in our previous trial. However, in our recent trial, the difference of severe wound complications was not significant and the incidence was the same in both groups. The analysed cohorts in our former trial were not identical to those investigated in the recent one. In the recent analysis, we made extensive efforts to have equivalent oncological groups of patients. We expanded the cohort of patients with open surgery to a total of more than 750 cases. In 80 of the patients operated on for colorectal cancer, we were able to find perfectly matched controls based on tumour location, tumour stage, mode of surgery and significant risk factors. We found identical oncological candidates and we could not find any significant differences in the oncological long-term outcomes. There was a trend for more successful results in the SILS group (5y-DFS 82 vs 70 % (*p* = 0.11), and 0 versus 4 local recurrences after surgery for rectal cancer (*p* = 0.117), respectively). It needs to be taken into account that almost 14% of the SILS intended operations were converted to open surgery. In an additional 6.2% of the patients, one additional trocar was used. The conversion rate is in line with data from other series on patients treated with minimally invasive surgery for colorectal cancer recruited in the beginning of the last decade [19]. The rather liberal conversion to open surgery in our cohort might in part be responsible for the 100% R0 resection rate and might also have slightly influenced the presented oncological candidate parameters.

Furthermore, in spite of the high rate of more than 50% teaching operations in both groups, SILS procedures were performed more frequently by more experienced consultants. Some degree of expert bias might have influenced the results as selection bias might have done. The patients that were not considered to be suitable for SILS for any reason were treated by open surgery. In spite of the fact that the median BMI was not significantly different between the two groups, it seems that patients with a BMI ≥ 30 mg/m^2^ were more likely to have been treated by open surgery, although this difference was not statistically significant. Regardless, the data indicate at least oncologic non-inferiority of SILS in comparison with open surgery in selected patients.

Our data also shows that SILS is not exclusively successful when performed by laparoscopic experts only. A substantial amount of these procedures had been done by residents or by newly qualified consultants with limited experience in minimally invasive colorectal surgery under the supervision of an expert surgeon. This suggests that the SILS technique was thoroughly integrated in the surgical training in our hospital. It is important to note that the residents were not in the beginning of their laparoscopic training. Before doing the first colorectal procedure, they had gained minimally invasive surgery experience by having performed up to 200 laparoscopic general surgery cases like cholecystectomies, appendectomies and hernia repairs. Colorectal cancer procedures and SILS can be included in the surgical residents’ teaching programmes.

One disadvantage of SILS is that the duration of the surgery tends to be longer, although the difference is 35 min. The median durations failed to reach significance due to the wide range of the values.

### What is the future relevance of SILS for the treatment of colorectal cancer?

Based on the recent literature, SILS has no clear benefit over MLS in the treatment of colorectal cancer. Two recent systematic reviews and meta-analyses found better oncological outcomes, lower complication rates, less pain, earlier recovery and less blood loss when comparing SILS with multiport laparoscopy [20, 21]. However, a majority of trials included in these reviews were non-randomized cohort trials with a high risk of both selection and expert bias. MLS however is easier. The unrestricted option of trocar placement does not only enable a more comfortable exposure and dissection but might also be helpful to achieve perfect specimens more easily and to improve oncological outcomes.

The next generation of robotic surgery in the future will enable surgeons to do single-incision surgery in a more comfortable setting by having a 3-dimensional view, adequate triangulation and facilities to comfortably suture intracorporal anastomoses [22, 23]. These robotic systems will provide the opportunity to choose the best site for the incision considering postoperative pain, acceleration of recovery and minimizing the risk of incision hernia while still harvesting a perfect oncological specimen. This may result in SILS being discussed as an attractive alternative to multiport laparoscopic surgery in the future.
